# Severe Hepatocellular Injury With Transaminases > 20,000 IU/L in Suspected Repeated Drug Ingestion Despite Undetectable Serum Levels

**DOI:** 10.7759/cureus.105189

**Published:** 2026-03-13

**Authors:** Vismay Patel, Rubba S Khan, Ajantha Rangasamy, Rehan Shah

**Affiliations:** 1 Internal Medicine, Hudson Regional Hospital (HRH) Bayonne University Hospital, Bayonne, USA; 2 Rheumatology, Hudson Regional Hospital (HRH) Bayonne University Hospital, Bayonne, USA

**Keywords:** acetaminophen toxicity, acute-on-chronic pancreatitis, alt (alanine aminotransferase), ast (aspartate aminotransferase), repeated supratherapeutic ingestion

## Abstract

Acetaminophen toxicity is a leading cause of acute liver injury and is typically evaluated using serum acetaminophen levels and the Rumack-Matthew nomogram. However, this approach does not apply to repeated supratherapeutic ingestion or delayed presentation, where levels may be low or undetectable despite severe liver injury. We report a 38-year-old woman with alcohol use disorder who presented with epigastric pain, nausea, and vomiting and was found to have acute-on-chronic pancreatitis and marked hepatocellular injury. Her liver enzymes rapidly worsened, peaking at aspartate transferase (AST) 22,966 IU/L and alanine aminotransferase (ALT) 1,498 IU/L, while the serum acetaminophen level remained <10 µg/mL. Further history revealed repeated ingestion of approximately 9 g of acetaminophen daily for four to five days prior to admission. Intravenous N-acetylcysteine was initiated despite the undetectable level. Liver enzymes subsequently improved, and the patient remained hemodynamically stable without encephalopathy. This case highlights that an undetectable acetaminophen level does not exclude severe toxicity and emphasizes the importance of early empiric treatment when clinical suspicion is high.

## Introduction

Acetaminophen toxicity is one of the most common causes of acute liver injury worldwide and remains the leading cause of acute liver failure in many countries [1,2]. Risk assessment traditionally relies on the Rumack-Matthew nomogram, which correlates serum acetaminophen concentration with time since ingestion to guide treatment decisions [3]. However, this tool is not applicable in cases of repeated supratherapeutic ingestion or delayed presentation, where serum levels may be low or undetectable despite significant hepatocellular injury [4,5]. In such situations, clinical history, laboratory trends, and risk factors such as chronic alcohol use are more reliable than serum drug levels alone. We present a case of severe hepatocellular injury with transaminases exceeding 20,000 IU/L in a patient with suspected repeated drug ingestion despite an undetectable serum acetaminophen level, highlighting the limitations of reliance on serum levels alone.

## Case presentation

A 38-year-old woman with a history of alcohol use disorder, depression, anxiety, and chronic pancreatitis presented on February 8, 2026, with two days of sharp epigastric pain radiating to the back, associated with nausea and multiple episodes of non-bloody, non-bilious vomiting. The patient reported a prior diagnosis of chronic pancreatitis approximately two years earlier during evaluation for recurrent abdominal pain, which was attributed to chronic alcohol use. She reported drinking approximately five shots of liquor daily for the previous four months and acknowledged intermittent heavier use in prior years, with recurrent episodes of alcohol-related abdominal pain.

On physical examination, the patient was alert and oriented but appeared uncomfortable due to abdominal pain. Vital signs were stable. Abdominal examination revealed epigastric tenderness without guarding or rebound. There was no jaundice, ascites, or stigmata of chronic liver disease.

Key laboratory trends during hospitalization are summarized in Table 1.

**Table 1:** Key laboratory values during hospitalization. WBC: white blood cell; BUN: blood urea nitrogen; AST: aspartate aminotransferase; ALT: alanine aminotransferase; INR: international normalized ratio; LDH: lactate dehydrogenase

Parameter	Admission	Peak/lowest	Last available	Reference range
WBC (K/µL)	11.1	10.4	5.4	4.0-11.0
Hemoglobin (g/dL)	14.3	10.5	10.5	12.0-16.0
Platelets (K/µL)	216	119	120	150-400
Sodium (mmol/L)	134	129	129	135-145
Potassium (mmol/L)	3.2	2.7	3.6	3.5-5.0
BUN (mg/dL)	5	<2	<2	7-20
Creatinine (mg/dL)	0.6	0.5	0.5	0.6-1.2
AST (IU/L)	2,325	22,966	749	10-40
ALT (IU/L)	216	1,498	381	7-56
Total bilirubin (mg/dL)	1.3	2.0	1.2	0.1-1.2
INR	1.39	1.46	1.46	0.9-1.1
LDH (U/L)	-	17,422	370	140-280
Lipase (U/L)	2,188	2,188	-	0-160
Magnesium (mg/dL)	1.4	1.3	1.6	1.7-2.2
Phosphorus (mg/dL)	2.9	1.9	1.9	2.5-4.5
Acetaminophen level (µg/mL)	<10	<10	<10	10-30 (therapeutic)

Initial laboratory testing revealed acute pancreatitis with a lipase of 2,188 U/L. Liver function tests showed significant hepatocellular injury with aspartate aminotransferase (AST) 2,325 IU/L and alanine aminotransferase (ALT) 216 IU/L. Total bilirubin was 1.3 mg/dL, and international normalized ratio (INR) was 1.39. White blood cell count was 11.1 K/µL. Serum acetaminophen level was <10 µg/mL. Electrolyte abnormalities included hypokalemia, hypomagnesemia, and hypophosphatemia.

**Figure 1:**
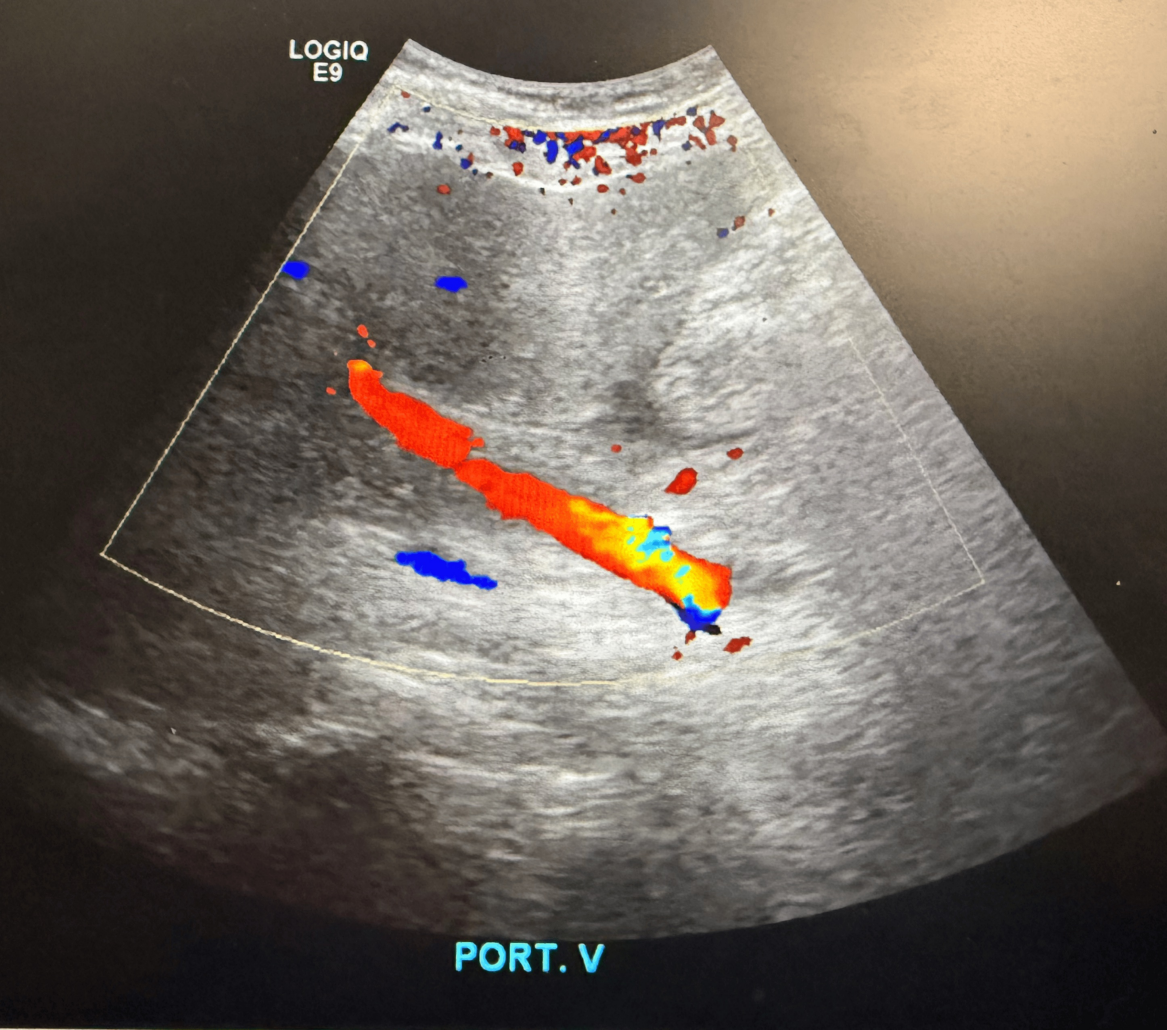
Doppler ultrasound of the liver demonstrating a patent portal vein with preserved hepatopetal flow and no evidence of portal vein thrombosis or obstructive pathology.

**Figure 2:**
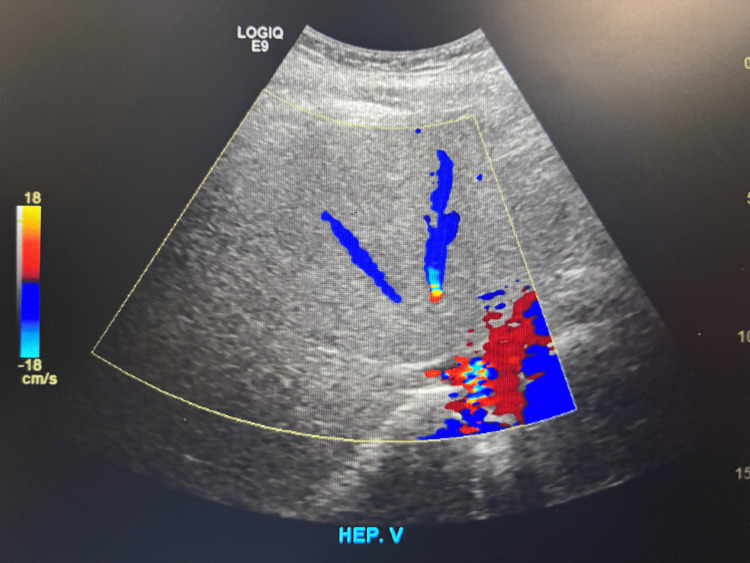
Doppler ultrasound of the liver demonstrating a patent hepatic vein with normal directional flow and no evidence of thrombosis or outflow obstruction.

**Figure 3:**
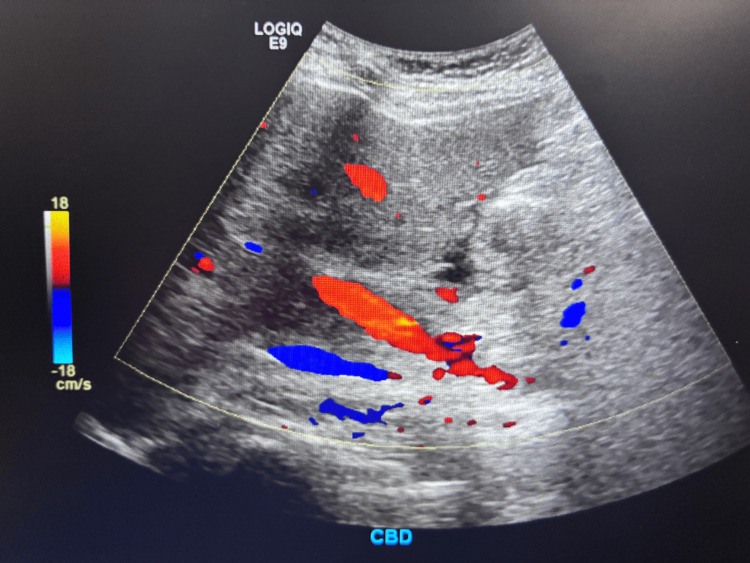
Ultrasound image demonstrating a normal-caliber common bile duct without dilation or obstructive pathology.

CT of the abdomen and pelvis demonstrated findings consistent with acute-on-chronic pancreatitis without necrosis, along with pancreatic calcifications and two rim-enhancing fluid collections near the pancreatic tail measuring 3.0 × 3.8 and 2.4 × 2.5 cm, consistent with pseudocysts. Abdominal ultrasound showed no gallstones or cholecystitis but revealed hepatomegaly with increased echogenicity consistent with hepatic steatosis. Doppler ultrasound demonstrated patent portal vein, hepatic vein, and common bile duct (CBD) with normal flow and no evidence of thrombosis or obstructive vascular pathology (Figures 1-3).

Magnetic resonance cholangiopancreatography (MRCP) was not performed during hospitalization because cross-sectional imaging and Doppler ultrasound demonstrated no biliary obstruction or vascular abnormality, and the patient’s clinical course improved with conservative management. The patient was managed conservatively with aggressive intravenous fluid resuscitation using lactated Ringer’s solution, bowel rest, antiemetics, and intravenous analgesia, with improvement in abdominal pain and tolerance of diet advancement.

During hospitalization, her liver enzymes rose dramatically, with AST peaking at 22,966 IU/L and ALT at 1,498 IU/L. Lactate dehydrogenase was markedly elevated at 17,422 U/L. The peak INR was 1.46, and total bilirubin peaked at 2.0 mg/dL. Viral hepatitis panel was negative, and HIV testing was negative.

Given the marked transaminase elevation, a more detailed medication history was obtained. The patient reported taking three 500 mg acetaminophen tablets every six hours, totaling approximately 9 g daily for four to five days prior to admission.

Despite the undetectable acetaminophen level, intravenous N-acetylcysteine was initiated for suspected drug-induced liver injury. Following treatment, her liver enzymes began to decline. On day two of treatment, AST decreased to 749 IU/L and ALT to 381 IU/L, with continued improvement during hospitalization. She remained hemodynamically stable and did not develop encephalopathy or require intensive care.

Electrolyte abnormalities including hypokalemia and hypomagnesemia were corrected with replacement therapy. She was monitored for alcohol withdrawal and received thiamine, folic acid, and multivitamin supplementation. No withdrawal symptoms were observed.

On February 11, 2026, the patient insisted on leaving the hospital. After discussion with the medical and gastroenterology teams regarding the risks and benefits of continued hospitalization, she initially agreed to stay. However, later that evening, she chose to leave against medical advice after signing the appropriate documentation.

Prior to discharge against medical advice, the patient was advised to follow up with gastroenterology and primary care for further evaluation including repeat liver function testing and consideration of additional workup such as triglyceride levels, IgG4 testing, and MRCP if clinically indicated.

## Discussion

Acetaminophen toxicity is one of the leading causes of acute liver failure worldwide [1,2]. Risk assessment is typically based on the Rumack-Matthew nomogram, which is applicable only in cases of single acute ingestion with a known time of exposure [3]. However, this approach is not reliable in cases of repeated supratherapeutic ingestion or delayed presentation [4,5].

Acute severe transaminase elevation is most commonly caused by ischemic hepatitis, viral hepatitis, or drug-induced liver injury [6,7]. In many countries, acetaminophen toxicity remains the most frequent cause of acute liver failure [1,8].

In cases of repeated supratherapeutic ingestion, serum acetaminophen levels may be low or undetectable at presentation despite significant hepatic injury [4,5,9]. This occurs because the drug may already be metabolized, while the toxic metabolite has initiated hepatocellular damage. In such situations, clinical history, transaminase levels, and risk factors such as chronic alcohol use are more informative than serum drug concentration alone [10].

This patient presented with extreme transaminase elevation and a negative acetaminophen level. Viral hepatitis testing was negative, and Doppler ultrasound demonstrated patent hepatic and portal veins without thrombosis, reducing the likelihood of vascular or obstructive causes. Further history revealed repeated supratherapeutic ingestion of acetaminophen for several days prior to admission, strongly suggesting drug-induced hepatocellular injury.

N-acetylcysteine remains the cornerstone of treatment for acetaminophen toxicity. Current guidelines recommend empiric initiation when there is significant transaminase elevation and suspected ingestion, even if serum levels are undetectable [2,11]. In this case, early initiation of N-acetylcysteine was associated with clinical stability and improvement in liver enzymes. This case highlights the limitations of relying solely on serum acetaminophen levels and emphasizes the importance of clinical judgment and early empiric treatment in suspected repeated ingestion.

## Conclusions

Severe hepatocellular injury can occur despite undetectable serum drug levels in cases of repeated supratherapeutic ingestion. Clinical history and laboratory trends should guide management decisions. Early empiric treatment with N-acetylcysteine is appropriate when clinical suspicion is high, even in the absence of measurable drug levels.
